# Annotations

**Published:** 1909-02-20

**Authors:** 


### Annotations.

### Mr. Bernard Shaw on the Medical Profession.

At a well-attended meeting of the Medico-Legal Society on Tuesday, Mr. G. Bernard Shaw advocated, with the manifest approval of many of his audience, the "socialisation" of the medical profession. His principal points were the following: The doctor is forced by competition to be a tradesman, who sells "cures" for what he can get. The average doctor is "appallingly and humiliatingly poor," and consequently a dangerous man. He dare not be "scientifically honest" with his patients, who would change their doctor if he ventured to tell them the truth and show them their ignorance. But the doctor is as honest as he can afford to be. The doctor shows much skill in adapting himself to the delusions of the patient, and as soon as he gets into general practice unlearns much of the hygiene which his hospital training has taught him. On a wholly unscientific plane the doctor does surprisingly well. It is thoroughly wrong that a doctor's income should be reduced by his own efficiency, and that by the advances of preventive medicine many doctors should be ruined. A bishop is not expected to blow the organ, but a doctor is expected to do any trumpery work, however great his skill. Under the competitive system men of transcendent ability waste their time on trivialities, and duffers are called upon to undertake work which is beyond them. The "socialisation" of the profession would remedy this. Socialism in the profession has already made strides, as is shown by the ever-growing number of medical men in the public service. The position of the medical offi[illegible]er of health is extremely enviable. Not being [illegible]pendent on the whims of his patients he alone [illegible] be independent and scientific in his pursuit o[illegible]ygiene. His success is judged by his vital statistics. There is much need for the intelligent application of statistical science to public health. This is only possible in the public service. Compulsory hygiene is spreading, and doctors are assuming rights over children to the exclusion of the rights of parents. This is well, but these powers cannot be exercised by individual tradesmen. The State must step in. Socialism would give every medical man the alternative of working honourably in the public service independent of the quackery which the demands of patients almost impose on private practitioners. In the discussion which followed Sir T. Clifford Allbutt and Sir Victor Horsley expressed a qualified approval of many of Mr. Shaw's propositions.

### Manchester Infirmary and Women Doctors.

Manchester is proud, and rightly proud, of its new Royal Infirmary, and the city which used to claim to be "first in commerce, first in art," may be congratulated on attaining the front rank in hospital construction and arrangement. For the present, however, the new development is disturbed by an acute controversy regarding an aspect of the question which is so prominent just now in other directions, namely, the position and opportunities of women. To judge from statements in the local papers it appears that the Medical Board is in favour of the admission of women as house physicians and house surgeons and that many members of the Board of Management take the same view. Yet, for some reason or other, the plan does not go forward and there is a general feeling of uncertainty. In such circumstances the advocates of the women's cause are pressing their case with their usual zeal and vigour. This has goaded the opposition into activity, and the local newspapers have been thick with contradictory letters and statements. In the meantime the Board hesitates, and having decided in favour of the appointments does not propose to carry the resolution into action. "Structural difficulties" and the need for a reorganisation of the domestic staff constitute, it is said, insuperable difficulties to the admission of women as medical officers, at least in the meantime. It is impossible not to sympathise with a Board of Management beset by the clamour of rival partisans, but the Board will do well to remember that the Infirmary exists neither to favour nor to oppose the claims of women, but to promote the welfare of the sick poor and to advance the knowledge of medicine and surgery. With this principle kept well in view there ought to be no difficulty in reaching and adhering to a clear and confident decision.

### The Fate of the Polyclinic.

Everyone interested in the post-graduate movement in London must feel glad that the Chenies Street institution, usually known as the Polyclinic, has weathered the financial crisis which recently threatened its continued existence. It has been known for a long time that the premier post-graduate college in the Metropolis did not pay its way on a commercial basis, and that private benefactions alone made it possible to carry on its work; and towards the close of last year it was very generally rumoured that the Polyclinic was about to close its doors. Indeed, the Council itself decided, as it appears from a published statement of Dr. Theodore Williams, the chairman, that such a step would be necessary to avoid incurring debts which it might not be possible to discharge. Two factors taken together enabled the Council to abandon this decision in January, and to proceed forthwith to arrange a new list of cliniques and lectures. These two factors are an increase in the amount of members' subscriptions, and a remission of interest for two years on his mortgage by Sir Jonathan Hutchinson, together with a donation of £100 per year for the same period. In congratulating the authorities on the solution of their difficulties, and in expressing the hope that it may be an effective one, we cannot close our eyes to the fact that Sir Jonathan's benefaction is merely a temporary one. An institution which cannot pay interest to the mortgagees of its home can hardly be said to be a financial success. Still it is much to be thankful for that the way is clear, at any rate for two years; and that, besides its routine of teaching, the Polyclinic will continue for that length of time to house the collections which Sir J. Hutchinson has laboured so untiringly to make. We have great satisfaction in wishing a new lease of life to the institution, and a greater measure of prosperity.

